# Bruton’s tyrosine kinase inhibitor associated localized extremity edema and erythema

**DOI:** 10.1016/j.jdcr.2024.10.001

**Published:** 2024-10-16

**Authors:** Shannon Meledathu, Paul Chu, Tatyana Feldman, Andre Henri Goy, Rachel Kushner Rosenstein

**Affiliations:** a Hackensack Meridian School of Medicine, Nutley, New Jersey; b Bridge Dermpath, Tarrytown, New York; c John Theurer Cancer Center, Hackensack Meridian Health, Hackensack, New Jersey; d Department of Medical Sciences, Hackensack Meridian School of Medicine, Nutley, New Jersey; e Department of Internal Medicine, Center for Discovery and Innovation, Hackensack Meridian School of Medicine, Hackensack University Medical Center, Hackensack, New Jersey

**Keywords:** Bruton's tyrosine kinase inhibitor, case reports (general dermatology), drug reactions, edema, leukemia, lymphoma, oncology

## Introduction

Bruton’s tyrosine kinase (BTK) inhibitors, such as ibrutinib, zanubrutinib, and acalabrutinib, are used to treat non-Hodgkin lymphoma, among other hematologic malignancies and inflammatory diseases. BTK inhibitors are associated with multiple dermatologic complications, including edema, which has not been extensively clinically characterized.^1^ Herein, we report 2 cases of individuals on BTK inhibitors with localized erythema over an extremity that progressed to severe pitting edema in one case.

## Case report

### Case 1

**Fig 1:**
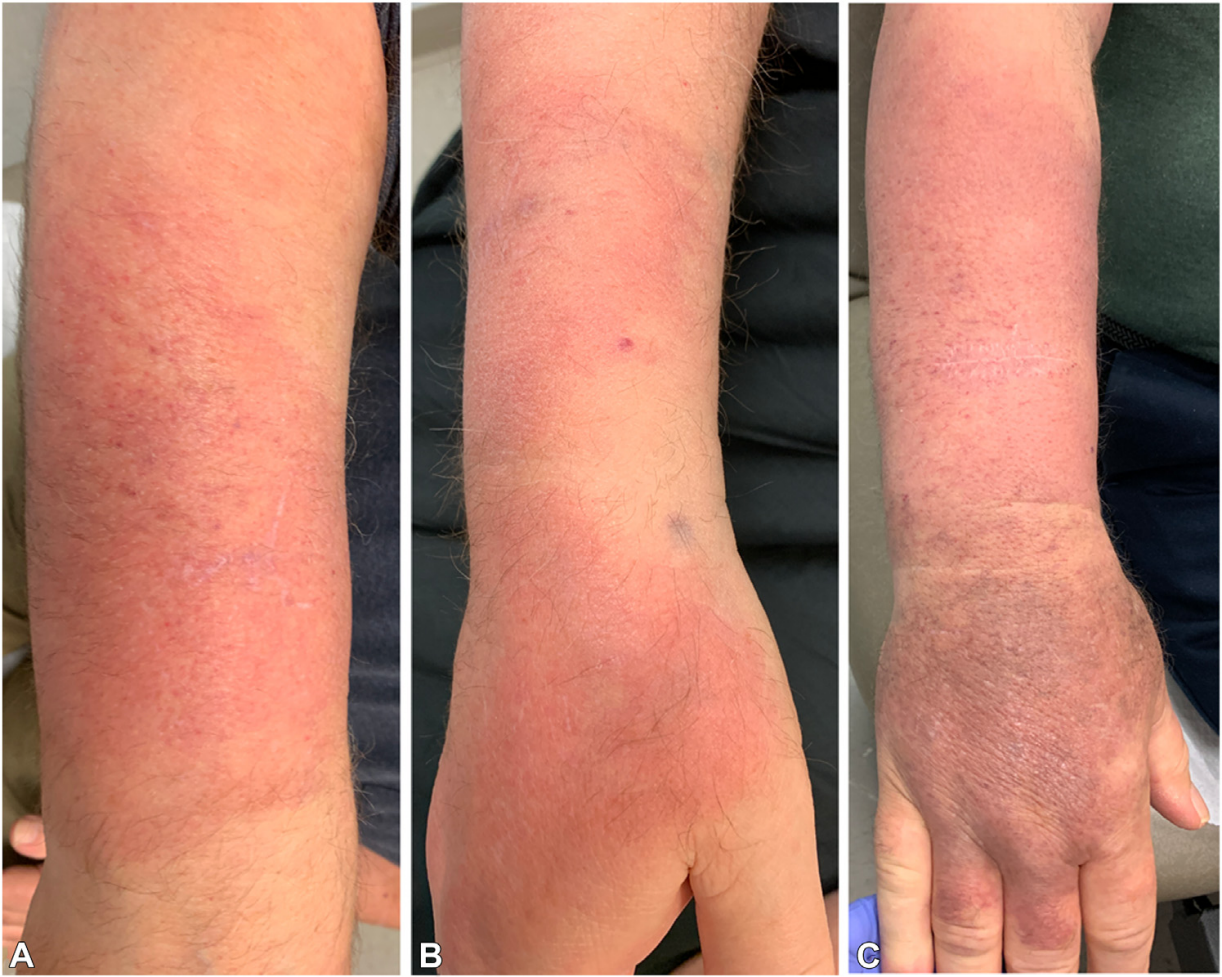
Case 1. Ibrutinib-induced edema and erythema in a 78-year-old man. **A,** The patient’s right forearm depicting a well-demarcated erythematous-violaceous plaque with irregular borders with telangiectasias at the initial presentation. **B,** The patient’s right forearm and hand 2 months after initial presentation. **C,** The patient’s right forearm and hand exhibiting worsening edema 18 months after initial presentation.

**Fig 2:**
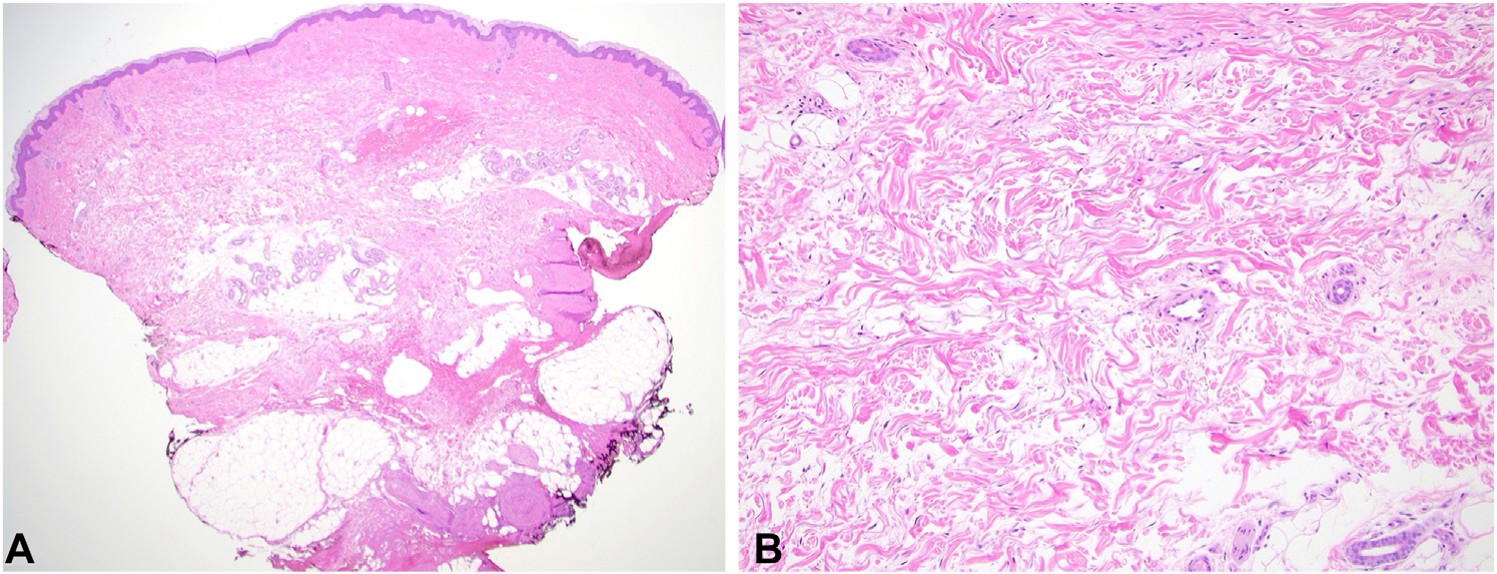
**A,** Hematoxylin and eosin sections show a segment of skin extending to the subcutis with absence of inflammatory infiltrate. There are loosely arranged reticular collagen bundles with widened spaces between them. (Original magnification: ×20.) **B,** On a higher magnification, loosely arranged collagen bundles are surrounded by wide spaces between them because of edema. (Original magnification: ×100.)

A 78-year-old man with a history of marginal zone lymphoma presented with new asymptomatic erythematous plaques after 4 years on ibrutinib. He had completed 3 years of rituximab therapy 2 years before presentation. On examination, there was a large (∼15 cm) erythematous-violaceous edematous plaque over the right forearm with a well-demarcated irregular border within which were telangiectasias and few white linear macules (Fig 1, *A*). There was a ∼4 cm erythematous plaque over the left forearm. Punch biopsy histopathology from the right arm revealed solar elastosis and telangiectasias accompanied by few extravasated erythrocytes. Ibrutinib was discontinued because of hypertension and the skin findings. After 2 months, the erythema progressed distally over the right forearm to the dorsal aspect of the hand accompanied by increasing edema (Fig 1, *B*). The left forearm lesion resolved. Because of concern for deep venous thrombosis, duplex ultrasound was done, which revealed no signs of superficial or deep venous thrombosis. Magnetic resonance imaging showed nonspecific edema/inflammatory change within the superficial soft tissue of the forearm and dorsal aspect of the hand with involvement of the superficial fascial planes. Magnetic resonance angiogram and venogram showed no abnormalities. The patient initially reported slight improvement on prednisone (60 mg and then 100 mg, tapered over 5 and 12 weeks, respectively), but soon pitting edema developed over the forearm and hand. An incisional biopsy was done revealing loosely arranged reticular collagen bundles with widened spaces between them because of edema and no significant inflammatory infiltrate (Fig 2, *A, B*). Eighteen months after initial presentation, the pitting edema and discoloration worsened and were associated with burning pain (Fig 1, *C*). Venous stasis was managed with elevation and compression.

### Case 2

**Fig 3:**
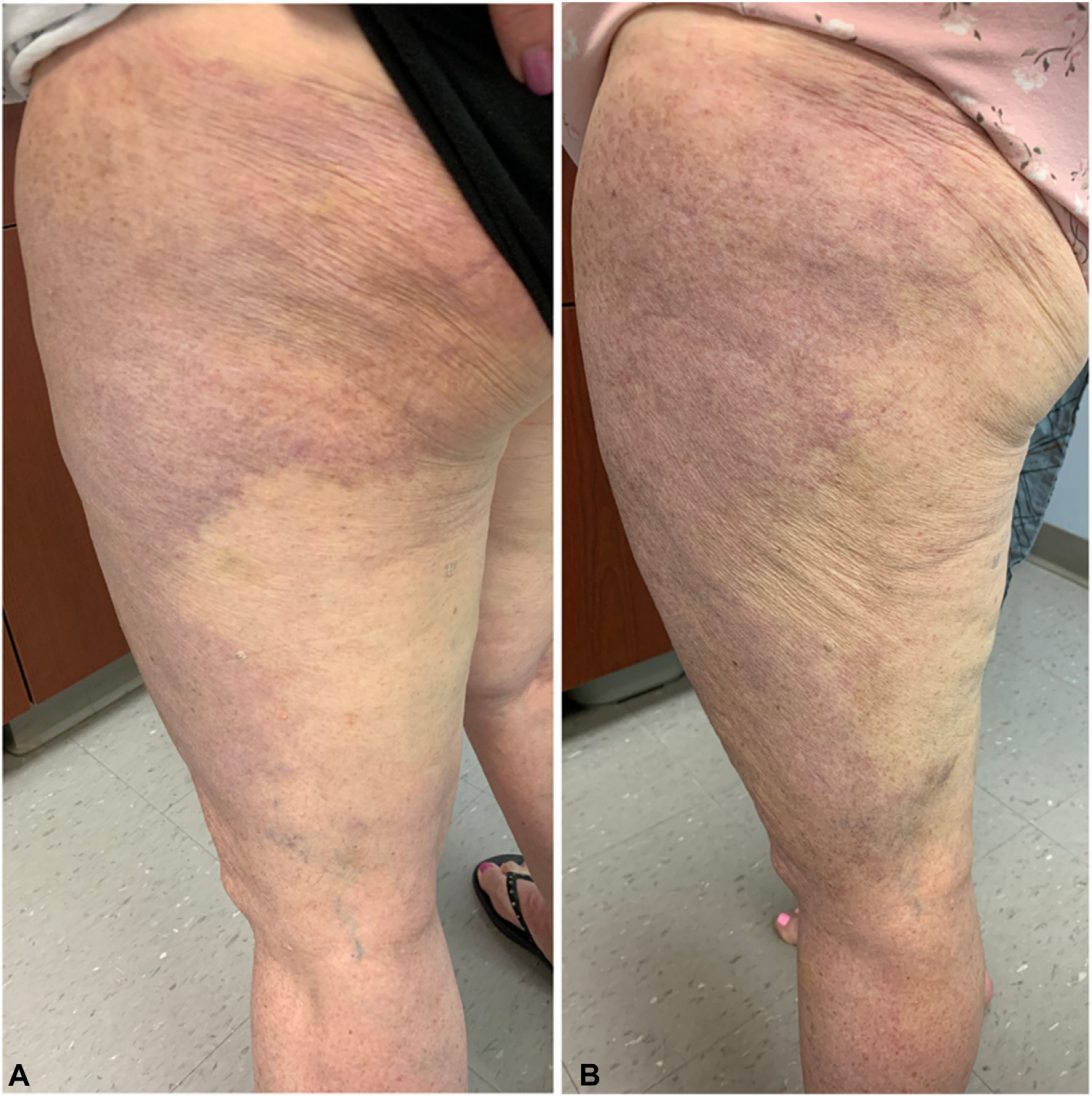
Case 2. Acalabrutinib-induced edema and erythema in a 64-year-old woman. **A,** The patient’s left thigh and buttocks depicting a well-demarcated violaceous plaque with irregular borders with superimposed telangiectasias at the initial presentation. **B,** A view of the patient’s left thigh and buttocks 2 years later showing progression and involvement of the lower portion of the leg.

A 64-year-old woman with chronic lymphocytic leukemia presented with a large violaceous-hyperpigmented plaque on the left thigh and buttocks associated with increased sensitivity to touch after 2.5 years on acalabrutinib. She had completed 6 months of rituximab 2 years prior. On examination, there was a well-demarcated plaque with an irregular border with superimposed telangiectasias and subtle associated scale (Fig 3, *A*). Histopathology revealed solar elastosis and telangiectatic vessels without an inflammatory infiltrate. Duplex ultrasound showed no superficial or deep venous thrombosis. Magnetic resonance imaging indicated no inguinal lymphadenopathy. Computed tomography angiogram showed no abnormalities. The patient was instructed to utilize compression stockings. While on acalabrutinib over the next 2 years, the plaque progressed distally to involve the lower portion of the leg (Fig 3, *B*). The extent of edema remained mild. During perioperative periods when acalabrutinib was held for several weeks, the patient noted that the erythema diminished.

## Discussion

BTK inhibitors are frequently associated with adverse cutaneous reactions. Ibrutinib can inhibit other kinases (Src, Tec, and epidermal growth factor receptor [EGFR]), which can result in adverse reactions because of its off-target effects.^2^ An integrated safety analysis found peripheral edema in 22% of patients receiving BTK inhibitors, but these studies did not clinically characterize the presentation.^3^ Second generation BTK inhibitors may have fewer off-target effects. A randomized phase 3 trial showed that edema was reported at ≥10% higher incidence among patients on ibrutinib compared to zanubrutinib.^4^

Most cutaneous adverse effects of BTK inhibitors can be treated without discontinuation of BTK inhibitor therapy, however, management of edema is challenging.^1^^,^^5^ The mechanisms that promote development of edema are unknown, but some have proposed a role for off-target inhibition of phosphoinositide 3-kinase, which can inhibit lymphangiogenesis and may produce capillary leakage, or platelet derived growth factor receptors, which can decrease interstitial fluid pressure and increase capillary-to-interstitium transport.^6^, ^7^, ^8^ A prior report of localized extremity edema because of BTK inhibitor therapy found no significant improvement with dose reduction in one case and slight improvement after discontinuation in the second case.^9^ In our series, case 1 continued to experience worsening edema after discontinuation of ibrutinib, whereas the skin changes in case 2 were overall well tolerated and acalabrutinib was continued. This patient had progression of body surface area involvement, but the grade of the edema did not worsen. Also, although both patients had previously been on rituximab therapy, and studies do report its association with peripheral edema, typically early on in treatment, to our knowledge, rituximab has not been associated with localized peripheral edema resembling the cases here.^10^, ^11^, ^12^, ^13^

Our cases exhibit the variability in disease course of localized extremity edema and erythema in the setting of BTK inhibitor therapy. Both cases began with a well-demarcated, irregularly bordered, erythematous-violaceous plaque that presented proximally and progressed distally, but only one case progressed to debilitating edema that persisted despite discontinuation of therapy. Overall, this presentation is quite distinct from typical peripheral edema. Although early findings were clinically suggestive of an inflammatory etiology such as a pigmented purpuric dermatosis, or less likely angiosarcoma, later findings in case 1 were more suggestive of eosinophilic cellulitis or deep vein thrombosis. As histopathology showed no evidence of inflammation or neoplastic cells, imaging was done to evaluate for thrombosis or obstruction that could produce this irregular edema with duplex ultrasound and magnetic resonance imaging. Although age may have contributed to their risk of venous insufficiency, they had no clinically significant cardiac, hepatic, or renal insufficiency or hypoalbuminemia and their clinical presentation was not typical of standard peripheral edema. Awareness of the initial disease presentation, which is clinically suggestive of an inflammatory dermatosis, will allow early initiation of compression therapy, and potentially improve clinical outcomes and quality of life in these patients. Further research into the pathogenesis of this adverse effect is necessary to improve treatment options for these patients.

## Conflicts of interest

Dr Goy received Grant/Research Support: PI (research to institution)-Acerta, AstraZeneca, Genentech, Janssen, Janssen Global Services, Kite Pharma and Pharmacyclics; is a Stock/shareholder in COTA, Alloplex, Resilience, Genomic Testing Cooperative; Consulted with Michael J. Hennessey; and served on the Advisory/Board-Kite, Janssen Biotech, Pharmacyclics LLC; Astrazeneca; COTA, Resilience, Genomic Testing Cooperative; Consulting Faculty/Speaker for Michael J Hennessey Associates, INC, Physicians Education Resource, LLC, Society of Hematologic Oncology, 3rd GCC Hematology, OncLive; served on the Scientific Advisory Board for Alloplex, Vincerx; Astrazeneca. Dr Feldman has worked in Consultancy, Advisory Board, Speakers Bureau; *ADC Therapeutics*: Consultancy, Advisory Board; *Astrazeneca*: Consultancy, Advisory Board, Research Funding; Celgene/*BMS*: Speakers Bureau, Research Funding; *Corvus*: Research Funding; *Daiichi Sakyo*: Consultancy, Advisory Board, Research Funding; *Eisai*: Research Funding; *Genmab*: Consultancy, Advisory Board, Research Funding; *Janssen Biotech*, Inc: Speakers Bureau; *Juno*: Research Funding; *Karyopharm*: Consultancy, Advisory Board; *Kite, a Gilead Company*: Consultancy, Advisory Board; *Kymera*: Research Funding; *MorphoSys*: Consultancy, Advisory Board; Research Funding; *Pharmacyclics LLC*: Speakers Bureau; Portola: Research Funding; *Poteligeo*: Speakers Bureau; *SecuraBIO*: Consultancy, Advisory Board; *Seattle Genetics*: Speakers Bureau, Research Funding; *Takeda*: Speakers Bureau; *Tessa*: Research Funding; *Genomic Testing Cooperative*: Other: Equity holder in privately-traded company. Remaining authors declare no conflicts.
